# Upstream ORFs as Regulators of Viral Replication

**DOI:** 10.3390/ijms27219821

**Published:** 2026-11-01

**Authors:** Yulia Agapkina, Anna Mashkovskaia, Sofia Shekhtman, Sergey Korolev, Tatiana Kikhai, Andrey Anisenko, Marina Gottikh

**Affiliations:** 1 Chemistry Department, Lomonosov Moscow State University, 119992 Moscow, Russiasssonnya@gmail.com (S.S.);; 2 Belozersky Institute of Physico-Chemical Biology, Lomonosov Moscow State University, 119992 Moscow, Russia; mashav012345@gmail.com (A.M.);; 3 Faculty of Bioengineering and Bioinformatics, Lomonosov Moscow State University, 119992 Moscow, Russia

**Keywords:** uORF, uPeptides, viruses, translation regulation

## Abstract

Upstream open reading frames (uORFs) are widespread regulatory elements located in the 5′-untranslated regions of eukaryotic and viral mRNAs. Once considered rare exceptions to the canonical scanning model, uORFs are now recognized as actively translated features present in approximately half of human transcripts, frequently initiating at non-canonical start codons. In eukaryotes, uORFs fine-tune protein synthesis in response to cellular stress by modulating ribosome reinitiation, as exemplified by the stress-dependent regulation of ATF4 and GCN4. In viral genomes, uORFs serve as compact, evolutionarily conserved modules that optimize protein stoichiometry, coordinate expression with the host cell state, and support persistent infection across diverse viral families through *cis*-regulation of translation. Beyond this, uORF-encoded peptides (uPeptides) can possess independent biological activities, influencing virulence, tissue tropism, and immune modulation. In this review, we systematize the available data on the dual functionality of uORFs as *cis*-regulatory elements of translation and as sources of bioactive uPeptides in viruses. Deciphering the full spectrum of uORF-mediated mechanisms may reveal novel layers of viral replication control and uncover potential targets for antiviral development, although, as discussed here, the available evidence for such applications is still largely limited to proof-of-principle mutagenesis.

## 1. Introduction

For a long time, the prevailing view in molecular biology of eukaryotic translation held that cellular mRNAs typically contain only a single open reading frame (ORF). Although the 5′-untranslated region (5′-UTR) of an mRNA may harbor multiple AUG codons, translation initiation begins at the first start codon in a suitable nucleotide context [1,2]. This context has come to be known as the Kozak sequence. According to the canonical scanning model, the ribosome binds at the 5′-end of the mRNA and migrates along the leader region until it reaches the first AUG in an optimal context, where it initiates translation, while upstream non-optimal start codons are either ignored or support only weak initiation [1,2].

For decades, this paradigm shaped approaches to the study of translation and genome annotation, even though exceptions had already been described in the 1980s and 1990s—viral and cellular mRNAs carrying regulatory upstream ORFs (uORFs) within their 5′-UTR [3,4,5,6]. Such cases might have been dismissed as isolated exceptions; however, the picture changed dramatically in 2009 with the advent of ribosome profiling, a technique that enables precise mapping of ribosome positions on mRNA across the entire transcriptome [7]. Its application in yeast revealed that translated uORFs are widespread throughout the transcriptome and that their translation levels are modulated by culture conditions, thereby confirming their regulatory potential. Subsequent studies in human and other mammalian cells demonstrated that actively translated uORFs are found in roughly half of all mRNAs, with a considerable fraction of these uORFs initiating from non-canonical start codons (non-AUG) [7,8,9,10]. These early estimates have since been refined: a consolidated, high-resolution map of human RNA translation assembled from more than a thousand ribosome profiling datasets confirms the widespread translation of uORFs in human cells [11], and the current community-curated catalog of non-canonical open reading frames lists over seven thousand entries, among which uORFs and uORFs overlapping the main reading frame form the largest class [12].

Importantly, uORFs are not a peculiarity of cellular transcriptomes, and they are of particular interest in viruses, which are the subject of this review. The same ribosome profiling approaches applied to infected cells have shown that viral mRNAs are similarly rich in translated upstream reading frames: a recent pan-viral survey identified 4208 previously unannotated ORFs in 679 viral genomes, a substantial fraction of which are uORFs [13]. Because viral genomes are under strong selective pressure toward compaction, such elements offer an economical way of adding a regulatory layer to viral gene expression without increasing genome size. Through this layer, viral uORFs set the stoichiometry of viral proteins translated from polycistronic or bicistronic mRNAs, couple viral translation to the stress state of the infected cell, and, in a number of cases, encode peptides that act as determinants of virulence and tissue tropism. The functional weight of these elements is illustrated by the fact that inactivation of a single viral uORF may reduce the yield of infectious particles by two to three orders of magnitude [14] or abolish the infectivity of the virus altogether [15]. The regulatory logic of uORFs summarized below for cellular mRNAs is therefore presented here mainly as a framework for the subsequent analysis of viral uORFs.

**Figure 1:**
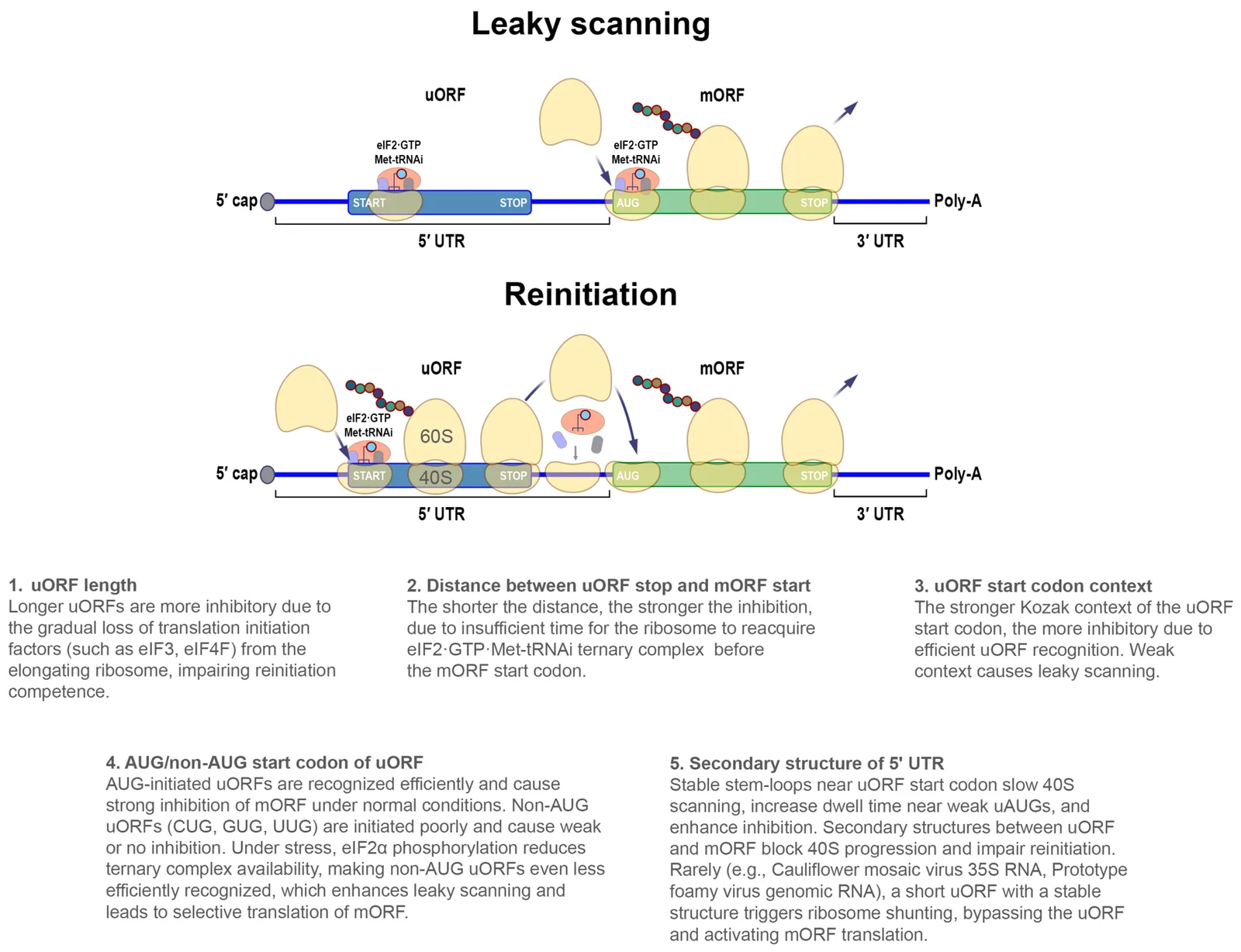
General structure of eukaryotic and viral mRNA and key factors determining the effect of uORFs on downstream mORF translation. Translation initiation begins with the recruitment of the 43S preinitiation complex comprising the 40S ribosomal subunit in association with eIF1, eIF1A, eIF3, and the ternary complex (eIF2–GTP–Met-tRNAi), to the 5′-terminal m^7^G cap through the eIF4F complex (eIF4E, eIF4G, eIF4A) together with eIF4B. The complex then scans the 5′-UTR downstream until it encounters a start codon in a favorable Kozak context. Initiation can occur either at the start codon of the mORF (**top panel**) or at the start codon of a uORF (**bottom panel**). If the ribosome, during leaky scanning, reaches the start codon of the mORF and initiates translation there, synthesis of the main protein proceeds independently of reinitiation. If, however, initiation occurs at the start codon of a uORF, the ribosome translates the uORF and terminates at its stop codon, after which synthesis of the mORF product becomes dependent on reinitiation. Whether reinitiation can occur depends on the number and length of uORFs, the strength of their Kozak context, the distance between the uORF stop codon and the mORF start codon, and whether the uORF overlaps the mORF. A more detailed analysis of these factors is provided in Section 2.

Today, uORFs are actively studied as *cis*-regulatory elements of main open reading frame (mORF) translation. In most cases, such elements act as repressors of mORF translation, since the probability of reinitiation at a downstream start codon of the main open reading frame decreases after the ribosome has translated a uORF (Figure 1) [1,2]. However, cases of more fine-tuned regulation have also been described, in which a uORF or a cluster of several uORFs can act as a repressor under certain conditions while, under others, conversely stimulating translation from the mORF, as has been shown for GCN4 in yeast and ATF4 in mammals [16,17,18].

Despite significant progress in understanding the mechanisms by which uORFs function as cis-regulatory elements of translation, it is important to recognize that their functionality is not limited to this role. uORFs themselves are translated sequences, and their peptide products may possess functions of their own. For a long time, peptides encoded by uORFs (uPeptides) were regarded primarily as unstable, rapidly degraded byproducts of the mechanism regulating mORF translation, devoid of independent biological activity. In recent years, however, this view has changed. Through a combination of ribosome profiling, mass spectrometry, comparative genomics, and functional approaches to studying uPeptides, it has been shown that at least some uPeptides can be stable, evolutionarily conserved, and perform functions of their own [19,20]. In human cells, uPeptides have been identified as ligands of HLA complexes, which confirms their expression and persistence in the cell [19], and several individual examples have been assigned defined molecular functions: ASDURF, encoded by a uORF of the ASNSD1 mRNA, is a prefoldin-like subunit of the PAQosome [21]; MIEF1-MP, encoded by a uORF of the MIEF1 mRNA, associates with the mitoribosome and affects mitochondrial protein synthesis [22]; and uPEP2, encoded within the PRKCH transcript, inhibits kinases of the PKC family [23]. These host-derived examples are mentioned here briefly and for one specific reason: they establish the proof of principle that a short reading frame in a 5′-UTR can give rise to a stable, functionally active product, and they define the experimental logic—conservation of the amino acid rather than the nucleotide sequence, detection of the endogenous peptide, and loss-of-function of the start codon—that is now being applied to viral uORFs, where, as discussed in Section 3.2, uPeptides can act as virulence and tropism determinants. Unfortunately, despite the growing number of studies devoted to the functions of such peptides, our knowledge in this area remains fragmentary, and there appears to be a growing need to reconsider the view on uORFs and the approaches to studying them.

uORFs merit particular attention in the context of studying viruses, their impact on the viral life cycle, infection productivity, and virus–host cell interactions. Because viral genomes tend toward miniaturization, the presence of such elements within them adds an additional layer of regulation to these processes (Figure 1). In this review, we have systematized the available data on the prevalence of uORFs in RNA and DNA viruses, their influence on viral infection through *cis*-regulatory properties, and through the independent functions of their peptide products.

## 2. uORFs as *cis*-Regulatory Elements of Eukaryotic Translation

Regulation of translation is one of the key mechanisms enabling a rapid and selective cellular response to changes in the intracellular and extracellular environment. Unlike transcriptional control, translational regulation allows the level of protein biosynthesis to be adjusted promptly, without the need for transcription and processing of mRNA. This level of control becomes especially important under stress conditions, e.g., nutrient deficiency, hypoxia, or oxidative stress, when the cell must reduce overall protein synthesis while preserving translation of a specific set of mRNAs, as well as during viral infections.

Among the *cis*-regulatory elements of the 5′-UTR, uORFs occupy a special place, and they are currently regarded as one of the central mechanisms of selective control of translation initiation. As noted above, such elements are present in approximately half of human transcripts, and in most cases a uORF reduces the efficiency of mORF translation [10].

The mechanism of *cis*-regulation of translation involving uORFs is based on the fact that the scanning small ribosomal subunit (40S) encounters an upstream start codon before the main one (Figure 1). If initiation occurs at the uORF, the subsequent fate of the ribosome depends on several parameters: the number of uORFs within a single transcript, their length, the distance from the 5′-cap to the upstream start codon, the context of the start codon, the distance between the uORF stop codon and the mORF start codon, whether the uORF overlaps the main start codon, and the ability of the 40S complex to remain on the mRNA and reinitiate translation [24]. In general, repression of mORF translation is enhanced by an increase in the number and length of uORFs, as well as by a reduction in the distance between the uORF stop codon and the mORF start codon. Particularly pronounced suppression is observed when a uORF overlaps the beginning of the main coding region [24]. These patterns are consistent with the fact that, during elongation on the uORF, the ribosome loses some initiation factors, and the probability of reinitiation is determined by the rate at which these factors are recruited after uORF translation is completed (Figure 1).

At the same time, the functional significance of uORFs is not limited to simple inhibition of mORF translation. These elements can act as flexible regulators of mORF translation, either suppressing it or finely redirecting translation in favor of stress-adaptive proteins. Under normal conditions, uORFs often serve as a barrier to mORF translation, whereas when the cellular state changes, they can ensure selective expression of adaptive proteins, for example, through the availability or activity of translation initiation factors [25,26,27].

uORFs acquire particular significance precisely under conditions of cellular stress. When adverse factors act on the cell, eIF2α is phosphorylated by stress-activated kinases, which reduces the availability of the ternary complex eIF2–GTP–Met-tRNAi and leads to a general suppression of translation initiation [28]. Against this background, however, some transcripts containing several regulatory uORFs become translated more efficiently. This activation is explained by the fact that, when the level of the ternary complex decreases, the probability of reinitiation at the second and subsequent inhibitory uORFs declines, allowing ribosomes to reach the mORF start codon while bypassing the inhibitory uORFs [29,30].

The most studied example of such regulation is ATF4 in mammals. The 5′-UTR of ATF4 mRNA contains two uORFs, the second of which overlaps the mORF, making it a strong inhibitory element. Under normal physiological conditions, after translating the first short uORF, the ribosome rapidly rebinds the ternary complex and reinitiates translation at the downstream inhibitory uORF, thereby preventing initiation at the main coding sequence. Under stress accompanied by eIF2α phosphorylation and reduced availability of the ternary complex eIF2–GTP–Met-tRNAi, rebinding of the complex is slowed, allowing ribosomes to bypass the start codon of the inhibitory uORF and reinitiate translation at the mORF start codon. As a result, ATF4 synthesis increases, despite the overall reduction in the intensity of translation within the cell [31,32,33]. A similar situation is observed in the regulation of GCN4 biosynthesis in yeast upon a shift to low-nutrient media. The 5′-UTR of GCN4 mRNA contains four uORFs, none of which overlaps the mORF; nevertheless, under normal conditions, owing to the high probability of reinitiation at the inhibitory uORF2–uORF4, ribosomes produce virtually no mORF product, whereas under nutrient deficiency they are more likely to skip these uORFs, increasing the chance of reinitiation at the mORF [17].

Another interesting example of fine-tuned regulation is the autoregulation of the translation initiation factor eIF5 synthesis. Upon interacting with the eIF2–GTP–Met-tRNAi ternary complex and recognizing the start codon, eIF5 induces the hydrolysis of the GTP bound to eIF2, thereby facilitating the transition from mRNA scanning to the assembly of the 80S ribosomal initiation complex. eIF5 influences start codon selection: specifically, the higher its concentration, the greater the probability of initiating translation at a start codon in a suboptimal context. This principle underlies the factor’s autoregulation. The 5′-UTR of its mRNA harbors a uORF that begins with an AUG start codon located in a suboptimal context. At low eIF5 concentrations, recognition of this weak start signal is less efficient, so ribosomes skip the uORF and continue scanning to the mORF start codon. This leads to increased eIF5 synthesis. As the eIF5 concentration rises, the probability of initiation at the weak AUG of the uORF increases. In this case, translation of the uORF reduces the number of ribosomes reaching the mORF and suppresses eIF5 synthesis. Thus, the uORF in the 5′-UTR of eIF5 mRNA forms a negative feedback loop that maintains a relatively stable intracellular concentration of this factor [34].

Beyond these classical models, recent work has extended the picture in several directions. First, uORF-dependent control of mORF translation can be regulated by cellular metabolites, e.g., polyamines induce slowing of ribosomes and their queuing on the uORF in the mRNA of antizyme inhibitor 1 (AZIN1), which redirects initiation to an upstream non-AUG start site and blocks mORF translation [35]. Second, systematic analysis of nascent-peptide-dependent uORFs has demonstrated that the sequence of the uORF product itself, and not merely the position of its start codon, can determine the regulatory outcome through interactions of the nascent chain with the ribosomal exit tunnel and the peptidyl transferase center [36].

An aspect that is frequently overlooked is that uORFs affect not only the rate of mORF translation but also the stability of the transcript that carries them. Since a uORF stop codon is by definition located far from the 3′-end of the mRNA, its recognition may be interpreted by the cell as premature termination and commit the transcript to nonsense-mediated mRNA decay (NMD); consequently, part of the repressive effect attributed to uORFs is exerted at the level of mRNA abundance rather than of translation efficiency, and the two contributions can only be separated by measuring transcript levels in parallel with reporter output [37].

Thus, uORFs are not merely inhibitory elements of the 5′-UTR but constitute a multi-level system for fine-tuning translation. Their regulatory effect is determined by the 5′-UTR architecture, the efficiency of start-codon recognition, the ribosome’s reinitiation capacity, and the availability of key initiation factors, primarily the eIF2–GTP–Met-tRNAi ternary complex (Figure 1). Consequently, uORFs enable the cell to regulate the synthesis of specific proteins in accordance with its functional state.

## 3. uORFs in Viral Genomes

For viruses, uORFs are not merely a feature of the 5′-leader region of mRNA but an important tool for adapting to an extremely compact genome and to the need for precise dosing of protein synthesis at different stages of infection. Viral genomes, in fact, make extensive use of overlapping and non-canonical modes of expression, and modern ribosome profiling shows that uORFs occur across a wide range of viruses substantially more often than previously thought. In a large pan-viral study using massively parallel ribosome profiling [13], 4208 previously unannotated ORFs were identified across 679 viral genomes, including numerous uORFs, indicating that this level of regulation is systematic rather than episodic.

Some examples of uORFs in viral genomes and of translation regulation mediated by them have been described in a review [38]. The most direct effect of uORFs on viral infection consists of the control of translation of key viral proteins, following the principles described above. In many cases, uORFs suppress initiation at the mORF and thereby set the required stoichiometry of viral products. For example, in human cytomegalovirus (HCMV), a uORF in the 5′-UTR of the UL4 mRNA has been shown to inhibit translation of the mORF, and this effect is associated not merely with the presence of an upstream start codon but with the encoded uORF peptide itself and with ribosomal stalling during termination [39,40]. In Kaposi’s sarcoma-associated herpesvirus (KSHV), the ratio of the amounts of ORF35 and ORF36 proteins, which are encoded within a polycistronic mRNA and are important for viral pathogenesis, has been shown to be controlled by two uORFs located in the 5′-UTR of the mRNA [41,42]. Similarly, a uORF within the hepatitis B virus pregenomic RNA suppresses initiation at the main coding sequence, which enables controlled production of polymerase while bypassing protein synthesis of the main reading frame [43,44]. These examples demonstrate that uORFs in viruses act as elements for fine-tuning the expression of viral proteins.

The role of viral uORFs is particularly important under conditions of cellular stress associated with viral replication. For example, it was shown that in Ebola virus (family *Filoviridae*), a uORF located in the 5′-UTR of the mRNA of the L polymerase acts as a molecular switch, which is crucial for successful viral replication. Under normal conditions, immediately after viral entry into the cell, the uORF suppresses translation of the viral polymerase; however, as stress pathways in the cell are activated in response to viral infection, it instead promotes enhanced expression. Mutations within the uORF that disrupt its *cis*-regulatory functions lead to a 100–1000-fold reduction in the production of viral particles [14]. This underscores that, for the virus, the critical factor is not the immediate production of the replication machinery right after entry, but rather the expression of such a protein in coordination with the state of the cell.

Pan-viral data confirm that this logic is not limited to isolated examples. In the study [13], viral genes with pronounced ribosomal occupancy in their 5′-UTR formed a distinct cluster, interpreted as uORF-dependent regulated transcripts. After induction of eIF2α phosphorylation with sodium arsenite, the proportion of viral genes in which ribosomes were predominantly retained in the 5′-untranslated regions decreased from 37% to 19%, while the proportion of genes with primary occupancy on the main coding sequences increased from 63% to 81%. This means that under stress conditions, ribosomes more frequently bypass inhibitory uORFs in viral genomes and initiate translation at the mORF [13]. Consequently, uORFs can be regarded as universal switches linking viral expression to the host cell’s stress response.

It is noteworthy that, in a number of cases, uORFs ensure not only the suppression or switching of translation but also the buffering (stability) of the translation of major viral proteins. The second uORF in the 5′-UTR of HCMV UL4 mRNA contains a terminal diproline motif that stalls ribosomes by disrupting the activity of the peptidyltransferase center [45]. It is the diproline motif that ensures ribosome stalling, thereby protecting translation of the mORF from a sharp decline when ribosome loading decreases. In other words, under conditions mimicking stress and a reduction in the overall efficiency of translation initiation, the downstream product is synthesized more stably than would be expected under simple constitutive repression. This is particularly important for viruses, since an infected cell is almost always in a state of translational stress; consequently, uORFs that cause ribosomal stalling may help the virus maintain synthesis of critical proteins even during global inhibition of host translation [46].

The functions of uORFs in viral genomes are not exhausted by *cis*-regulation of translation of proteins encoded in the main reading frame. In a number of cases, the peptide products of uORFs themselves contribute to virulence and tropism [47,48,49]. However, since the mechanisms by which such uPeptides affect the cell and the virus itself—unlike the mechanisms of translation regulation—differ significantly across different viruses, it is not possible to present a generalized concept of their mode of action. Such mechanisms will be examined using specific examples below.

From an evolutionary perspective, uORFs align with the general viral strategy of maximizing the use of limited genomic space. Overlapping genes are widespread among viruses, with the extent of overlap linked to constraints on genome size. In this context, uORFs offer an advantageous compromise: they enable the addition of a new layer of translational regulation without significantly increasing genome size, while in some cases also allowing for the synthesis of a small peptide crucial for a successful viral infection. This is precisely why uORFs are found in both RNA and DNA viruses, often proving essential for efficient viral replication and spread [50].

In summary, uORFs represent a widespread and evolutionarily conserved mechanism of translational regulation that is of particular importance in the context of viral infection. Their functional role extends far beyond the mere inhibition of mORF translation. uORFs act as dynamic sensors of the cellular state, integrating signals from stress response pathways and responding to the availability of initiation factors to fine-tune viral gene expression. They regulate the stoichiometry of viral protein synthesis and the rate of replication, enable the virus to adapt translation to stressful conditions within the infected cell, and, in some instances, encode functionally active peptides that influence virulence, viral progeny output, and tissue tropism. The dual nature of uORFs serving simultaneously as *cis*-regulatory elements and as templates for the synthesis of functionally active peptides renders them versatile modules of viral pathogenicity. Subsequent sections will examine specific examples of translational *cis*-regulation involving viral uORFs and the functions of the peptides they encode, thereby illustrating the diversity of strategies viruses employ to optimize the infection process. All examples discussed below are listed in Table 1.

### 3.1. Influence of Viral uORFs on Viral Replication via cis-Regulation of Translation

#### 3.1.1. Retroviridae

Retroviruses are enveloped viruses with a positive-sense single-stranded RNA genome of approximately 7000–12,000 nucleotides, the life cycle of which is based on reverse transcription of the genome and integration of the resulting DNA copy into a host chromosome. The canonical genome comprises the *gag*, *pol* and *env* genes flanked by long terminal repeats (LTRs), which are formed during reverse transcription and contain the promoter and enhancer elements used for transcription of viral RNAs by cellular RNA polymerase II; complex retroviruses such as human immunodeficiency virus (HIV) encode in addition the regulatory proteins Tat and Rev and the accessory proteins Nef, Vif, Vpr and Vpu [58]. Two features of this organization are directly relevant to the present discussion. First, all viral mRNAs are produced from a single primary transcript by alternative splicing, so that several proteins have to be expressed from a limited set of bicistronic and polycistronic mRNAs. Second, the ratio between regulatory and structural proteins must be maintained within narrow limits. Both constraints make the 5′-leader regions of retroviral mRNAs an obvious site of translational control.

It is currently known that the uORFs within the retroviral genomes perform a *cis*-regulatory function, and independent functions of the encoded uPeptides have not been studied for any of them. The presence of such uORFs allows for the regulation of ribosome access to the mORFs, which is particularly important for retroviruses characterized by compact genomes, multiple splicing variants, and the need to strictly maintain the ratio of regulatory to structural proteins. In some cases, retroviral uORFs suppress downstream translation, whereas in others, they promote it by stimulating reinitiation, with the net effect manifesting at the level of virus production [52,53]. Examples of such regulation are examined in greater detail below.

The bicistronic HIV-1 mRNA encoding Vpu and Env contains a minimal uORF consisting of only two codons (a start–stop element) and overlapping the Vpu start codon. The presence of this regulatory element not only reduces the level of Vpu synthesis but also stimulates Env production, as it enables ribosomes to reach the Env reading frame. Modification of the start or stop codon of this uORF reduced Env expression fivefold, while deletion of the uORF almost completely suppressed Env expression and rendered the virus non-functional. Importantly, this minimal uORF is conserved in 9 of the 11 consensus HIV-1 subtypes, and in the two remaining subtypes the absence of such a uORF is compensated for by weakening of the Kozak context of the Vpu start codon. This points to strong evolutionary pressure to preserve the uORF-dependent mechanism maintaining Env expression and, consequently, viral infectivity [15].

A similar mechanism operates in simian immunodeficiency virus (SIV). In its mRNA encoding Rev and Env, upstream AUG codons have been identified. uAUG4 strongly suppresses Rev translation but at the same time indirectly stimulates Env, since it reduces ribosome capture at the Rev start codon, which would otherwise interfere with reaching the Env AUG. In reporter systems, deletion of uAUG4 sharply enhanced Rev expression and disrupted the optimal Rev/Env ratio. Consequently, in SIV the uORF is required for successful viral replication, which is achieved by optimizing the ratio of Rev and Env levels [52,53]. Thus, the mRNA encoding Env employs similar mechanisms of Env expression control in both HIV-1 and SIV, involving regulation via uORFs.

A distinctive variant of uORF-dependent control has been described in prototype foamy virus (PFV). Its long 5′-UTR in genomic RNA contains several short uORFs and can fold into an extended hairpin that would by itself strongly impede linear scanning of the mRNA and translation of the Gag ORF. PFV has been shown to employ ribosomal shunting to overcome this obstacle. The ribosome loads onto the 5′-end of the mRNA, scans only the initial segment, translates one of the 5′-proximal uORFs, and then jumps over the central structured region of the leader and reinitiates translation at the downstream Gag-encoding ORF. Mutations that disrupt the uORF or the stem structure reduce Gag expression, whereas restoration and strengthening of stem complementarity, by contrast, increases the efficiency of ribosomal shunting. Thus, in PFV, short uORFs serve as key elements for launching Gag synthesis and, consequently, for the early stages of replication [53]. For PFV, it is particularly important that uORF-dependent ribosomal shunting apparently coordinates translation with other stages of the life cycle. The authors emphasize that the central structured region of the 5′-UTR of the genomic RNA contains regulatory elements involved in RNA dimerization and packaging into the virion, as well as in interaction with the tRNA primer; therefore, translation from such an mRNA while preserving the structure of its 5′-UTR may be an important mechanism for regulating the late stages of replication of this virus. In other words, the uORF in the PFV genomic RNA influences viral replication not only by controlling the amount of Gag but also by preserving the structure of the regulatory elements of the genomic RNA. Moreover, similar mechanisms of ribosomal shunting have been found in other primate foamy viruses, indicating that this strategy is conserved [53].

Recent data further show that in HIV-1, uORF-mediated regulation is not limited to canonical AUG codons. Ribosome profiling has revealed a broad set of uORFs with non-canonical start codons in the 5′-UTRs of viral mRNAs, particularly widespread in the full-length genomic RNA. These uORFs generally reduce translation of the main viral ORFs, but their inhibitory effect can be partially compensated for by the RNA helicase DDX3 and TAR-dependent mechanisms. Similar regulatory uORFs have also been found in HIV-2 and HTLV-1, further indicating the evolutionary fixation of such elements among retroviruses [51].

All the examples discussed above pertain to uORFs (upstream open reading frames) located within viral genomes. A conceptually different situation, which we will discuss separately here, arises when a virus regulates its own replication by influencing the expression of a cellular protein *via* a uORF located within a cellular mRNA. The relevant example is the uORF in the mRNA of APOBEC3G, a key HIV-1 restriction factor. APOBEC3G suppresses HIV-1 replication in two ways: through its deaminase activity, causing G-to-A hypermutations in viral cDNA, and through direct inhibition of reverse transcription [59,60,61]. To counteract APOBEC3G, HIV-1 employs the viral protein Vif, which promotes proteasomal degradation of APOBEC3G, prevents its incorporation into budding virions and also disrupts its translation [59,60,62]. Notably, this latter counteraction mechanism is directly linked to the presence of a uORF in the APOBEC3G mRNA. The uORF by itself negatively affects APOBEC3G biosynthesis, but in the presence of Vif its effect is enhanced, and the mRNAs themselves are redirected to stress granules in this case. Interestingly, upon deletion of the uORF or mutation of its start codon, the effect of Vif on APOBEC3G translation disappears completely [62]. This case illustrates that the uORF-based layer of translational control is accessible to viruses from both sides: viruses not only carry uORFs in their own genomes but can also exploit uORFs present in the mRNAs of host antiviral factors to increase viral progeny production or their own infectivity.

Thus, the influence of uORFs on retroviral replication is multifaceted. In HIV-1 and SIV, they primarily establish the correct ratio between regulatory and envelope proteins, thereby determining the rate of transition between life cycle stages and virion infectivity. In PFV, short uORFs orchestrate a ribosomal transition that simultaneously ensures Gag synthesis and preserves the structural elements of the genomic RNA required for subsequent replication. At the level of host-cell interaction, retroviruses also employ uORF-dependent mechanisms to suppress antiviral factors, as demonstrated by the Vif–APOBEC3G system. Therefore, uORFs should be regarded as critical regulatory nodes in retroviral replication.

#### 3.1.2. Herpesviridae

Herpesviruses are large enveloped viruses with a linear double-stranded DNA genome ranging from about 120 to more than 230 thousand base pairs, which circularizes in the nucleus and serves as the template for transcription and replication. Their genomes encode an extensive set of replication, structural, immunomodulatory and antiapoptotic proteins and support two alternative programs of existence: productive lytic infection and latency, during which the genome persists as an episome and only a limited set of transcripts and proteins is expressed [63,64]. For the present discussion, the relevant features of this organization are the extensive use of polycistronic transcripts, in which internal reading frames must be reached by scanning ribosomes, and the sharply different translational demands of the lytic and latent programmes.

In herpesviruses, uORFs represent important regulatory elements of their mRNAs that control the efficiency of viral protein translation and, consequently, influence the replicative cycle. Using the examples of HCMV and KSHV, it can be seen that uORFs can operate through different molecular scenarios: either suppressing translation of downstream ORFs or, conversely, providing ribosomes with access to an internal ORF of a polycistronic mRNA *via* reinitiation. In both cases, the net effect manifests at the level of synthesis of proteins required for efficient viral replication [39,41,42].

A classic example of the repressive action of a uORF in herpesviruses has been described for the mRNA of the HCMV glycoprotein gpUL4 (gp48). In this transcript, the critical regulatory element is uORF2, which suppresses translation not merely through the presence of an upstream AUG but owing to the specific features of its own coding sequence. Substitutions that disrupt the amino acid sequence of uORF2 relieve repression, whereas synonymous mutations preserve it. Moreover, the inhibitory effect requires uORF2’s own stop codon. This indicates that the peptide synthesized from uORF2 likely influences translation termination at this reading frame and thereby prevents ribosomes from progressing normally to the mORF. Although a direct quantitative relationship with virus production was not measured in this study, the high conservation of uORF2 among HCMV isolates points to its probable importance for the infectious cycle [39].

For KSHV, the regulation of protein expression from the ORF35–37 locus is the most thoroughly studied. This locus is transcribed as a functionally bicistronic mRNA encoding ORF35 (function unknown) and ORF36 (essential viral serine/threonine kinase). It has been shown that ORF36 expression does not depend on an IRES but requires 5′-cap recognition followed by scanning. The key mechanism involves two short uORFs in the 5′-UTR. uORF1 moderately suppresses ORF35 translation, whereas uORF2, located in a stronger Kozak context and overlapping the ORF35 start codon, plays a central role. Its translation allows a portion of ribosomes to bypass ORF35 and then reinitiate synthesis at ORF36. Disruption of uORF2 or weakening of its initiation increases ORF35 synthesis and sharply inhibits ORF36 expression, which leads to reduced infectivity, whereas artificial extension of uORF2 almost eliminates ORF36 translation. Thus, uORF2 acts as a switch that redistributes ribosomes between ORF35 and ORF36 [41,42].

A recent study by Harrington et al. showed that in KSHV, uORF-dependent regulation of translation depends not only on the uORF itself or the architecture of the mRNAs but also on the state of the ribosome [54]. The authors showed that during lytic KSHV replication, the amount of the N1-methyltransferase EMG1, a ribosome biogenesis factor, associated with the 40S subunit increases threefold. Simultaneously, an increase in the proportion of ribosomes carrying modified 18S rRNA is also observed. This indicates that KSHV induces the formation of specialized ribosomes with altered properties. Upon EMG1 knockdown, the abundance of viral mRNAs changed only slightly, but the production of late proteins decreased noticeably. Ribosome profiling showed that EMG1 knockdown leads to translation defects, which directly affect the yield of infectious viral particles [54].

Using luciferase reporters with the 5′-UTRs of the viral mRNAs encoding ORF28, ORF34 or ORF69, which contain regulatory uORFs, the authors showed that EMG1 knockdown reduces luciferase translation from such constructs. At the same time, the addition of wild-type EMG1 relieved this defect, whereas a catalytically inactive EMG1 mutant did not. Consequently, what matters for KSHV is not merely the structural role of EMG1 in 40S biogenesis but specifically its methyltransferase activity, which reduces ribosome occupancy at viral uORFs and thereby facilitates translation of the downstream mORFs of late lytic genes [54].

Thus, whereas earlier studies of HCMV and KSHV showed that uORFs themselves act as *cis*-regulatory elements determining whether translation of downstream ORFs will be suppressed or permitted, the recent study on EMG1 shows that herpesviruses are able to additionally reprogram the ribosome itself in order to change the rules by which these uORFs are read. In the case of KSHV, this makes it possible to selectively enhance translation of late viral proteins and increase the yield of infectious virions. Consequently, uORFs in herpesviruses should be considered not in isolation but as part of a broader system that includes virus-induced changes in ribosome composition and modifications.

#### 3.1.3. Coronaviridae

Coronaviruses are enveloped viruses with a positive-sense single-stranded RNA genome of approximately 22,000–36,000 nucleotides, one of the largest among known RNA viruses. The genomic RNA carries a 5′-cap and a poly(A) tail, resembles cellular mRNA and is translated directly upon entry into the cell. Its organization is conserved and can be represented as 5′-UTR–ORF1a/ORF1b–S–E–M–N–3′-UTR, with additional accessory ORFs located between the structural genes in different coronaviruses; these accessory proteins are generally dispensable for replication in cell culture but contribute to pathogenesis and to evasion of innate immunity. Of particular importance here is the fact that the 5′-UTR contains the leader sequence used in the discontinuous transcription of subgenomic mRNAs, together with conserved RNA structures required for replication, so that any uORF in this region is embedded in a functionally dense and highly structured element [65,66,67].

The 5′- and 3′-UTRs of the genomic RNA contain conserved RNA structures required for initiating replication, negative-strand synthesis, switching between replication and transcription, as well as for interaction with viral and cellular proteins. The 5′-UTR includes a leader sequence that plays an important role in the synthesis of subgenomic mRNAs, which are generated through the process of discontinuous transcription. From an evolutionary perspective, the coronavirus genome possesses notable plasticity. It is characterized by both point mutations arising during replication and recombination, which play an important role in the emergence of new variants and adaptation to new host species [65,66,67].

uORFs in coronaviruses are important *cis*-regulatory elements of the viral RNAs, capable of influencing translation efficiency and, through it, viral replication and persistence. According to Wu et al. [55], an AUG-initiated uORF is present in more than 75% of the analyzed coronavirus genomes from 38 reference strains and usually encodes a very short peptide of 3–13 amino acids. The function of the coronavirus uORF has been studied in most detail using the mouse hepatitis virus (MHV) model. In this virus, the uORF is located in the 5′-UTR of the genomic RNA, within a hairpin structure, and begins at nucleotide 99, whereas the main start codon of ORF1 begins at nucleotide 210. In vitro experiments have shown that this uORF can indeed be translated, since deletion of its stop codon results in the synthesis of a chimeric uORF–ORF1 protein [55].

It has been experimentally demonstrated that the uORF functions primarily as a repressor of ORF1 translation. Replacing the uORF start codon with the UAG stop codon while introducing a compensatory U112A mutation that preserves the uORF-harboring stem-loop resulted in an approximately 1.6- to 1.7-fold increase in ORF1 translation levels. Importantly, however, the uORF-null mutant virus remained viable and exhibited growth kinetics similar to those of the wild-type virus, so that no requirement for the uORF in replication was demonstrated. More interestingly, despite the absence of any effect of these mutations on viral replication efficiency, the virus was found to have restored the uORF by the fifth passage [55]. Taken together, these data establish a *cis*-regulatory effect of the MHV uORF on ORF1 translation and a selective advantage sufficient to drive reselection of the uORF during passage, but they do not establish a contribution to replicative fitness measurable in cell culture. Whether such a contribution exists in a more physiological setting, for example in mice, remains to be tested.

Another study reveals a different but conceptually related variant of uORF involvement in coronavirus biology—this time in the context of persistent infection. M. Hofmann et al. demonstrated that during the initial stages of bovine coronavirus (BCV) infection, the 5′-terminal regions of viral mRNAs are heterogeneous and hypervariable, whereas as persistence becomes established in cell culture, 5′-leader variants containing an AUG uORF emerge and are selected. The emergence of such a uORF leads to suppression of viral protein translation, which apparently reduces the cytopathic effect of the virus and is necessary for maintaining chronic infection [56].

Thus, uORFs in coronaviruses should be regarded as evolutionarily conserved translation regulators that act on at least two levels. First, in viruses such as MHV, the uORF restricts ORF1 translation and thereby likely helps maintain an optimal balance of replication proteins. Second, the uORF that emerges and is selected during BCV persistence is capable of more broadly attenuating translation of viral mRNAs and thereby promoting long-term infection. In both cases, the uORF appears to function not as an incidental element of the 5′-UTR but as a regulated module. It should be stressed, however, that these two levels rest on evidence of different strength: for BCV, the selection of uORF-containing leader variants during the establishment of persistence is a directly observed phenotype, whereas for MHV a contribution to fitness is inferred from the reselection of the uORF during passage and not from a measured growth difference.

#### 3.1.4. Hepadnaviridae

Hepatitis B virus (HBV) belongs to the family *Hepadnaviridae* and possesses a small, partially double-stranded relaxed circular DNA (rcDNA) genome of approximately 3.2 kb. After entry into the nucleus, rcDNA is converted into covalently closed circular DNA (cccDNA), which serves as the stable nuclear template for all viral transcripts and underlies the persistence of HBV infection. Among these transcripts, the pregenomic RNA is of particular relevance here: it acts both as the template for reverse transcription and as the mRNA for the core protein that forms the capsid (gene C) and for the polymerase (gene P), the reading frames of which overlap and the products of which must be synthesized at a fixed ratio from a single transcript. The S gene, encoding the surface proteins, and the X gene, encoding the regulatory protein HBx, are transcribed from cccDNA as separate mRNAs [68,69].

For a long time, the question of how polymerase P is synthesized remained unresolved: there is no dedicated mRNA for this protein; instead, it is encoded exclusively within the bicistronic pregenomic RNA, in which ORF-P is located downstream of ORF-C, and the two frames overlap. The answer to this question emerged precisely from the study of the uORF within pregenomic RNA. A. Chen et al. established that its 5′-UTR contains a short uORF, designated C0, whose start codon is located upstream of the *C* gene, while its 3′-region overlaps with ORF-C. Importantly, this uORF is highly conserved across all HBV genotypes and even among other members of the genera *Orthohepadnaviridae*, which may indicate the importance of this uORF for viral replication [43].

Using a reporter system, the authors confirmed that translation initiates at the uORF start codon despite the weak Kozak context both in vitro and in HepG2 cells. The efficiency of initiation in this system was 20% of the level of initiation from ORF-C. Since the free C0 peptide could not be detected in these systems, it was proposed that uORF-C0 functions as a *cis*-regulator of translation [43].

A. Chen et al. demonstrated that uORF-C0 suppresses translation of the core protein from ORF-C, but stimulates polymerase P biosynthesis from ORF-P. Deletion of the AUG start codon in uORF-C0 resulted in increased core protein expression and reduced polymerase production. Conversely, strengthening the C0 start codon context reduced core protein translation to 4% of the control level but enhanced P polymerase biosynthesis [43]. These findings are consistent with a model in which a subset of ribosomes initiates translation at the C0 codon and, consequently, can no longer reach the core protein AUG start codon due to the overlap of these reading frames, yet remains capable of driving polymerase P biosynthesis through translation reinitiation at ORF-P. Since the uORF-C0 start codon is located in a weak context, this system makes it possible to maintain an optimal C:P protein ratio of 10:1, which is important for successful viral replication [43,44].

Thus, uORF-C0 in HBV should be viewed as a crucial regulatory module that ensures the correct ratio of C and P gene products, a factor essential for successful viral replication. Its primary functions can be summarized in three points. First, it restricts the synthesis of the core protein. Second, it helps direct a portion of ribosomes toward reinitiation at the downstream ORF-P. Third, by doing so, it contributes to maintaining the proper C-to-P ratio, without which efficient HBV replication is impossible.

### 3.2. Viral uORFs Encoding Functional uPeptides

From the examples described above, it becomes clear that uORFs are indeed a powerful tool for controlling the translational efficiency of viral genes, enabling the virus to achieve the optimal protein ratios required for successful replication, as well as to control the timing of their synthesis in accordance with the phases of the infectious cycle and the state of the host cell—which is particularly important under conditions of cellular stress that accompany any viral infection. However, it should be kept in mind that such uORFs are not only regulatory sequences within the mRNA but also possess coding potential themselves. In this section, we discuss a few examples of such viral uPeptides for which a role in the viral life cycle has been described. It should be noted that the small number of examples of uPeptides participating in viral replication does not indicate that this mechanism is rarely used by viruses; rather, it reflects the difficulty of studying the roles of such peptides and the fact that the idea that such peptides exist in cells began to develop relatively recently. We would also like to draw the reader’s attention to the fact that the uORFs discussed here are not devoid of cis-regulatory activity affecting downstream translation. Nevertheless, we have included them in this section and have not discussed them above because, in addition to this activity, the peptides they encode have been experimentally shown to possess independent functions. For each example, we subsequently specify which observations point to the functional activity of the peptide itself and which point to the cis-regulatory activity of the reading frame.

#### 3.2.1. Flaviviridae

Flaviviruses, including Zika virus (ZIKV), are enveloped, positive-strand RNA viruses. Their genomes contain a single main open reading frame (ORF) encoding all structural and non-structural proteins, which is translated as a single polyprotein and subsequently processed into functional proteins by viral and cellular proteases [70]. Recently, additional uORFs within the 5′-UTR of the ZIKV genome were identified using ribosome profiling. Asian/American strains encode two uORFs (uORF1 and uORF2), whereas African strains encode only one, which corresponds to a fusion of the two uORFs found in Asian/American strains. Both uORFs from Asian/American strains are translated in cell lines transfected with the corresponding reporter mRNAs, albeit at significantly lower levels than the main ORF due to their non-canonical start codons (0.80% and 4.13% of main ORF translation, respectively). Mutations introduced into the uORF1 start codon that abolished uORF1 expression (uORF1-KO) enhanced main ORF translation, whereas analogous mutations in the uORF2 start codon (uORF2-KO) reduced it, by roughly 30–40% in each case, which once again confirms the *cis*-regulatory function of uORFs in viral genomes [48].

Notably, in U251 cell culture, the uORF1-KO virus displayed superior replicative fitness relative to the American WT virus, as additionally confirmed by competition assays. This phenotype is most readily attributable to the inhibitory effect of the uORF1 on the main ORF translation. In stark contrast, infection of human cortical neurons and cerebral organoids with the uORF1-KO virus resulted in a severe defect, with viral titres decreased by 30- to 100-fold, raising doubts about this hypothesis. Further investigation into this phenomenon revealed that the 3.1 kDa peptide encoded by uORF1 itself acts as a virulence factor in neurons. This peptide, which is predicted to adopt an alpha-helical structure, induces rearrangement of the vimentin cytoskeleton. Notably, substitution of isoleucine 14 with proline (I14P) within this peptide prevented colocalisation of vimentin with the uORF1-derived peptide. It is well established that ZIKV infection is accompanied by substantial cytoskeletal rearrangements, including the formation of cytoskeletal elements around viral factories, so-called cytoskeletal cages; however, this was not observed in cells infected with the uORF1-KO virus. Therefore, expression of the uORF1 polypeptide likely facilitates vimentin collapse and thus contributes to the formation of the cytoskeletal cage in infected cells [48].

Unfortunately, the functional role of uORF2 in viral replication could not be unequivocally determined in this study, as the uORF2-KO virus reverted to the WT sequence after passage 1. This may indicate a role for the uORF2 start codon in the translation of the viral polyprotein [48] or point to a functional contribution of the uORF2-encoded peptide.

#### 3.2.2. Picornaviridae

Enteroviruses, belonging to the family *Picornaviridae*, comprise a large group of non-enveloped, positive-sense, single-stranded RNA pathogens of mammals. Their genome contains a single ORF and a 5′-internal ribosome entry site (IRES) element, which is essential for the efficient translation of the viral polyprotein in infected cells [71]. Interestingly, the IRES element of poliovirus type 1 and some other enteroviruses contains a conserved AUG start codon, which is followed by a short uORF that overlaps the main ORF. The coding potential of such uORFs has long been considered unlikely, since no protein product (6.5 to 9.0 kDa for different viruses) could be detected in infected cells. However, this view was challenged by the work of V. Lulla et al. Comparative analysis of over 3000 enterovirus sequences demonstrated conservation of the uORF and its encoded amino acid sequence across major enteroviruses, implying functional relevance for viral fitness for both the uORF and its peptide product. Ribosome profiling of echovirus 7 (EV7), poliovirus type 1 (PV1), or enterovirus A71 (EV-A71) infected RD cells confirmed ribosome occupancy at the uORF start codon in all cases; however, EV-A71 demonstrated markedly lower uORF translation efficiency. Additionally, expression of the uORF-encoded protein was confirmed by Western blotting for EV7 and PV1 [47].

The study by Lulla et al. demonstrated that this protein performs a specific function during enterovirus infection, particularly in gut epithelial cells—the primary site of viral entry. Using human intestinal organoids, they showed that the uORF-KO EV7 virus is attenuated compared to the wild-type virus at late stages of infection. Importantly, this effect was not observed in standard cell culture systems (RD cells), suggesting that its role is specifically linked to the gut epithelial cells. The uORF-encoded proteins of different enteroviruses contain a predicted transmembrane domain, and their membrane localization was experimentally confirmed by membrane fractionation and immunofluorescence microscopy, demonstrating that the protein associates with cellular membranes during infection. Together, these data suggest that this membrane-associated protein facilitates the release of viral progeny from infected cells at late stages of the infection cycle, thereby promoting viral spread within the intestinal epithelium [47].

#### 3.2.3. Arteriviridae

Porcine reproductive and respiratory syndrome virus (PRRSV), a member of the family *Arteriviridae*, is an enveloped positive-sense RNA virus. The 15 kb genome contains 10–12 ORFs, encoding more than 20 proteins. A recent study, which used ribosome profiling of PRRSV-infected cells to characterize changes in protein expression during infection, identified an additional uORF that is constitutively and highly translated. This uORF is present in North American PRRSV isolates, is highly conserved, and differs in only a small proportion of isolates. The AUG start codon is present in 558 of the 564 available genome sequences, and the remaining six contain the non-canonical start codons GUG, AUA, or ACG, suggesting that this reading frame may play an important role in the viral life cycle [57]. Since this reading frame encodes a very short peptide of only 10 amino acids, G. Cook et al. hypothesized that the encoded uPeptide is unlikely to be functional. Instead, they proposed that the strong conservation of this uORF among strains may confer a fitness advantage, for example, by modulating the translation of other ORFs [57].

However, T. Liu et al. later demonstrated that the uORF-encoded peptide is in fact functional [49]. Using a bioinformatic analysis of the 5′-UTR of the highly pathogenic PRRSV strain JXwn06, they identified two potential uORFs: uORF1 (spacer-less and therefore unlikely to be expressed) and uORF2, which encodes a 10-amino-acid peptide and corresponds to the uORF previously described by Cook et al. [57]. Notably, these uORFs are conserved not only among PRRSV strains but also across the major members of the family *Arteriviridae* [49].

Using a luciferase fusion reporter system, the authors showed that uORF2, but not uORF1, is translationally active. As reported for many other uORFs, uORF2 negatively affected expression from the downstream mORF start codon [49].

To assess the biological relevance of uORF2, the authors generated a mutant variant of the highly pathogenic PRRSV JXwn06 strain in which the uORF2 start codon was changed from AUG to the much weaker non-canonical start codon GUG. This mutant replicated as efficiently as the wild-type virus in MARC-145 cells and primary porcine alveolar macrophages (PAMs), indicating that the mutation does not affect PRRSV replication in vitro. In piglets, the mutant virus caused disease symptoms, with even more pronounced lung lesions than the wild type. In addition, it triggered a stronger intracellular immune response, accompanied by increased expression of the inflammatory cytokines TNF-α, IL-1β, and IL-6 both in vitro and in vivo [49].

Further experiments demonstrated that the short 10-amino-acid peptide encoded by uORF2 (MPRHLYCQEL) itself regulates the magnitude of the intracellular immune response. Expression of the inflammatory cytokine TNF-α in response to poly(I:C) treatment was significantly reduced in cells expressing a uORF2–GFP fusion protein, but not in cells expressing GFP alone [49]. Although the mechanism underlying this suppression of the pro-inflammatory response remains unclear, these observations suggest that even very short peptides encoded by uORFs can themselves possess intrinsic biological functions.

Thus, PRRSV uORF2 exemplifies the full trajectory from uORF discovery to functional uPeptide characterization. Initially predicted to be non-functional due to its short length, the 10-amino-acid peptide MPRHLYCQEL proved capable of modulating the intracellular immune response. This challenges the assumption that only long uPeptides can be biologically active and suggests that the pool of functional viral uPeptides may be larger than currently appreciated.

## 4. Conclusions

The prevalence of uORFs in eukaryotic and viral mRNAs and their functional significance are now well established. The discovery of these elements and the subsequent realization of their widespread occurrence across eukaryotes and viruses have provided new insight into the mechanisms of translational regulation. While cellular mRNAs were until recently viewed primarily as monocistronic templates translated from the first “strong” AUG codon within an optimal Kozak context, ribosome profiling has revealed that actively translated uORFs are present in approximately half of all human transcripts, with a significant proportion initiating at non-canonical start codons. It is now evident that the presence of such elements in eukaryotic mRNA enables cells to respond rapidly to changing conditions by modulating the biosynthesis of specific adaptive proteins through the *cis*-regulatory functions of uORFs.

uORFs perform similar functions in viral genomes, where they are particularly advantageous given the tendency of viruses toward genome miniaturization and the need for precise control over protein synthesis at various stages of infection. The examples discussed in this review demonstrate that viral uORFs are integral components of translational regulation rather than rare exceptions. uORFs determine viral protein stoichiometry and replication rates, allow the virus to adapt translation to stress within the infected cell, and, in some instances, facilitate the maintenance of persistent infections. At the same time, the strength of the evidence differs considerably from one example to another. For some uORFs, such as those of HIV-1, SIV, KSHV or Ebola virus, loss-of-function experiments establish a direct contribution to the production of infectious virus; for others, such as the uORF of mouse hepatitis virus, a *cis*-regulatory effect is firmly demonstrated while a contribution to replicative fitness remains inferred from conservation and from reselection during passage rather than measured. Keeping this distinction explicit, as we have attempted to do throughout and in Table 1, seems to us a precondition for the further development of the field.

A second conclusion concerns the dual nature of these elements. Alongside their *cis*-regulatory activity, the peptide products of several viral uORFs possess functions of their own, contributing to virulence, viral yield and tissue tropism. Here, however, a significant imbalance in research focus is evident. The vast majority of studies, including those cited above, concentrate on uORFs as *cis*-regulatory elements, for which a more or less universal methodology exists and which therefore naturally sustains the publication of new work. Far fewer studies address the independent functions of the encoded uPeptides, yet these few studies demonstrate that even very short peptides can possess intrinsic biological activity, as the ten-residue peptide of PRRSV illustrates. The reasons for this scarcity are objective: each uPeptide requires an investigative approach tailored to its specific function, a function that is unknown *a priori* and difficult to predict from a short peptide sequence alone. This situation calls for a re-evaluation of how uORFs are studied in the context of viral replication, and for the development of function-oriented approaches capable of systematically identifying uPeptides with independent activity in viral and cellular genomes.

Crucially, although the fundamental importance of uORFs for producing the major viral proteins in the correct stoichiometry or at the correct time in the viral cycle is well established, interfering with these *cis*-regulatory elements in practice is extremely difficult.

Finally, it is worth asking what practical use this knowledge may eventually find. Attempts to control viral replication through uORFs are still in their infancy, and none has so far advanced beyond proof of principle, but as our understanding of these elements expands, two directions become visible, both of which follow from the coding capacity of viral uORFs. The first rests on the simple fact that uPeptides are translated and that at least some of them are processed and presented on HLA class I molecules, where they elicit specific T cell responses; such sequences may therefore be considered as immunogens for vaccine design, and they are of particular interest precisely because they are absent from constructs designed on the basis of annotated reading frames alone. The second is to regard uPeptides themselves as targets for antiviral compounds. The feasibility of this approach will depend on the mechanism of action of each individual peptide, and the most realistic option at present appears to be the development of compounds that prevent a uPeptide from engaging its cellular partners. Thus, the ZIKV uORF1 peptide acts through a specific, sequence-dependent association with the vimentin network, which is lost upon a single I14P substitution; blocking such an association would be expected to attenuate the virus in the cell types where it matters. It should be emphasized, however, that even where the mechanism of action of a uPeptide is understood, the design of such inhibitors will be complicated by the absence of structural information on these peptides, by their small size and by the lack of well-defined binding pockets; for the same reasons, competing peptides or peptidomimetics may prove a more realistic modality than conventional small molecules. The immediate priority is therefore not medicinal chemistry but the structural and interactomic characterization of viral uPeptides, which at present is available for almost none of them. Such work would not only deepen our understanding of the molecular mechanisms of viral infection but would also provide the rational basis without which uORF-directed antiviral and vaccine strategies will remain at the level of proof of principle.

## Figures and Tables

**Table 1:** An overview of viral uORFs and their impact on viral replication.

Virus (Family)	uORF Localization	uPeptide	Effect on mORF Translation (*cis*-Regulation)	Function of the uPeptide	Main Experimental Approaches	Effect on Viral Replication and Level of Evidence	Ref.
HIV-1 (*Retroviridae*)	Bicistronic *vpu*/*env* mRNA; minimal two-codon start–stop uORF in the 5′-UTR; AUG; overlaps the *vpu* start codon	Not studied (2 codons)	Reduces Vpu synthesis and increases Env synthesis by allowing ribosomes to reach the *env* reading frame; mutation of the start or stop codon reduces *env* expression fivefold	Not studied	Reporter assays; site-directed mutagenesis; analysis of conservation in 11 consensus subtypes	Deletion of the uORF almost completely abolishes Env expression and renders the virus non-functional (demonstrated)	[15]
HIV-1, HIV-2, HTLV-1 (*Retroviridae*)	Genomic and spliced viral mRNAs; multiple uORFs with non-canonical (non-AUG) start codons; most abundant in the full-length genomic RNA	Several non-AUG uORF products, presented on HLA molecules	Generally reduces translation of the main viral ORFs; the inhibitory effect is partly compensated by the RNA helicase DDX3 and by TAR-dependent mechanisms	Elicit specific T cell responses; relevance for immune recognition rather than for replication	Ribosome profiling; reporter assays; immunopeptidomics; T cell assays	Effect on viral transcript expression shown; a requirement for replication has not been tested (proposed)	[51]
SIVmac239 (*Retroviridae*)	*rev*/*env* mRNA; upstream AUG codons in the 5′-UTR	Not studied	Strongly suppresses Rev translation and indirectly stimulates Env translation; deletion of uAUG4 sharply increases Rev expression and distorts the Rev/Env ratio	Not studied	Reporter assays; mutagenesis of upstream AUG codons	Required for an optimal Rev/Env ratio and thereby for efficient replication (demonstrated)	[52,53]
PFV (*Retroviridae*)	Genomic RNA; several short 5′-proximal uORFs within a long structured leader containing an extended hairpin	Not studied	Translation of a 5′-proximal uORF initiates ribosomal shunting over the structured part of the leader and reinitiation at *gag*; disruption of the uORF or of the stem reduces Gag expression	Not studied	Reporter assays; structure-guided mutagenesis of the uORF and of the stem; comparison with other primate foamy viruses	Required for efficient Gag synthesis and, indirectly, for preservation of the 5′-UTR elements used in dimerisation and packaging (demonstrated for expression, proposed for replication)	[53]
HCMV (*Herpesviridae*)	UL4 (gpUL4/gp48) mRNA; uORF2 in the 5′-UTR	22-codon peptide, acting in *cis* and not detected as a free product	Strong repression of mORF translation that depends on the amino acid sequence of the uORF2 peptide and on ribosome stalling at the terminal diproline motif; also buffers mORF output when initiation rates decline	Nascent-peptide-dependent stalling; no function outside the ribosome has been described	Reporter assays; synonymous versus non-synonymous mutations; cryo-EM of the stalled ribosome; kinetic modeling of translational buffering	Repression and stalling demonstrated; importance for the infectious cycle inferred from conservation among isolates (proposed)	[39,40,45,46]
KSHV (*Herpesviridae*)	Polycistronic ORF35–37 mRNA; uORF1 and uORF2 in the 5′-UTR; uORF2 has a stronger Kozak context and overlaps the ORF35 start codon	Not studied	uORF1 moderately suppresses ORF35; translation of uORF2 allows a fraction of ribosomes to bypass ORF35 and reinitiate at ORF36; artificial extension of uORF2 almost abolishes ORF36 translation	Not studied	Reporter assays; mutant viruses; ribosome profiling and EMG1 knockdown/rescue with a catalytically inactive mutant	Disruption of uORF2 inhibits ORF36 expression and reduces infectivity; EMG1-dependent ribosome occupancy at viral uORFs affects the yield of infectious particles (demonstrated)	[41,42,54]
MHV (*Coronaviridae*)	Genomic RNA; uORF at nucleotide 99 within a hairpin; present in >75% of analyzed coronavirus genomes	Predicted 3–13 residues; no peptide detected	Represses ORF1 translation; inactivation of the start codon with a compensatory mutation preserving the stem-loop increases ORF1 translation 1.6–1.7-fold	Not studied	Reverse genetics; in vitro translation; stop-codon deletion generating a uORF–ORF1 fusion; serial passage	The uORF-null mutant is viable and grows like the wild type in cell culture; the uORF is reselected by passage 5, so a fitness contribution is inferred from reselection only (proposed)	[55]
BCV (*Coronaviridae*)	5′-leaders of viral mRNAs; intraleader AUG uORF that emerges and is selected during the establishment of persistent infection	Not studied	Attenuates translation of viral mRNAs	Not studied	Sequencing of 5′-leader variants during persistent infection; reporter assays	Selection of uORF-containing leaders accompanies the establishment of persistence and reduced cytopathic effect (selection demonstrated; causal contribution proposed)	[56]
HBV (*Hepadnaviridae*)	Pregenomic RNA; uORF-C0 in the 5′-UTR; AUG in a weak Kozak context, initiation at ~20% of the ORF-C level; 3′-part overlaps ORF-C; conserved in all genotypes and in other *Orthohepadnaviridae*	Not detected	Suppresses core protein translation from ORF-C and stimulates polymerase synthesis from ORF-P by reinitiation; strengthening the C0 context reduces core translation to 4% of control	Not studied	Reporter assays in vitro and in HepG2 cells; deletion and context mutations of the C0 start codon	Maintains the C:P ratio of about 10:1 required for replication (demonstrated in reporter systems; inferred for the virus)	[43,44]
Ebola virus (*Filoviridae*)	mRNA of the L polymerase; uORF in the 5′-UTR	Not studied	Represses polymerase translation shortly after entry and promotes its expression once cellular stress pathways are activated, acting as a stress-dependent switch	Not studied	Reporter assays; recombinant viruses with mutated uORF; induction of the integrated stress response	Mutations disrupting the *cis*-regulatory function reduce the production of viral particles 100–1,000-fold (demonstrated)	[14]
ZIKV (*Flaviviridae*)	Genomic RNA; uORF1 and uORF2 in the 5′-UTR of Asian/American strains (African strains carry a single fused uORF); non-canonical start codons; translated at 0.80% and 4.13% of the mORF level	uORF1: 3.1 kDa, predicted α-helical; uORF2: not characterized	uORF1-KO increases and uORF2-KO decreases mORF translation, by roughly 30–40% in each case	The uORF1 peptide induces rearrangement of the vimentin cytoskeleton and contributes to the formation of cytoskeletal cages around viral factories; the I14P substitution abolishes colocalisation with vimentin	Ribosome profiling; reporter mRNAs; recombinant viruses; competition assays; cortical neurons and cerebral organoids; immunofluorescence	uORF1-KO virus: increased fitness in U251 cells but a 30- to 100-fold drop in titre in neurons and organoids (demonstrated, cell-type dependent); the role of uORF2 is unresolved, as the mutant reverted after the first passage	[48]
EV7, PV1, EV-A71 (*Picornaviridae*)	Genomic RNA; uORF starting at a conserved AUG within the IRES and overlapping the main ORF; conserved in >3000 enterovirus sequences	6.5–9.0 kDa depending on the virus; contains a predicted transmembrane domain	No substantial effect on polyprotein translation has been reported; the element is conserved at the amino acid rather than the nucleotide level	Membrane-associated protein that facilitates the release of viral progeny from infected cells at late stages of infection	Ribosome profiling of infected RD cells; Western blotting; conservation analysis; human intestinal organoids; membrane fractionation; immunofluorescence	The uORF-KO EV7 virus is attenuated in human intestinal organoids at late stages of infection, but not in RD cells (demonstrated, tissue-specific)	[47]
PRRSV (*Arteriviridae*)	Genomic RNA; uORF1 (spacer-less, not translated) and uORF2 in the 5′-UTR; AUG of uORF2 present in 558 of 564 genome sequences; conserved across major *Arteriviridae*	uORF2: 10 residues (MPRHLYCQEL)	uORF2 is constitutively and highly translated and negatively affects expression from the downstream mORF start codon	The 10-residue peptide reduces induction of TNF-α and limits the intracellular pro-inflammatory response	Ribosome profiling; luciferase fusion reporters; recombinant virus with an AUG → GUG substitution; infection of MARC-145 cells, primary alveolar macrophages and piglets	The mutant replicates as efficiently as the wild type in vitro and causes disease in piglets with more pronounced lung lesions and a stronger inflammatory response; no requirement for replication (*cis*-effect and peptide function demonstrated)	[49,57]

## Data Availability

No new data were created or analyzed in this study. Data sharing is not applicable to this article.

## Abbreviations

The following abbreviations are used in this manuscript:

5′-UTR	5′-Untranslated Region
BCV	Bovine Coronavirus
cccDNA	Covalently Closed Circular DNA
HBV	Hepatitis B Virus
HCMV	Human Cytomegalovirus
HIV	Human Immunodeficiency Virus
HTLV-1	Human T-Cell Leukemia Virus Type 1
IRES	Internal Ribosome Entry Site
KSHV	Kaposi’s Sarcoma-Associated Herpesvirus
LTR	Long Terminal Repeat
MHV	Mouse Hepatitis Virus
mORF	Main Open Reading Frame
ORF	Open Reading Frame
PFV	Prototype Foamy Virus
PRRSV	Porcine Reproductive and Respiratory Syndrome Virus
PV1	Poliovirus Type 1
rcDNA	Relaxed Circular DNA
SIV	Simian Immunodeficiency Virus
TAR	Trans-Activation Response Element
uORF	Upstream Open Reading Frame
uPeptide	Upstream Open Reading Frame-Encoded Peptide
ZIKV	Zika Virus
